# Sub-involution, Wiih Retro-flexion

**Published:** 1869-12

**Authors:** F. B. Norcom

**Affiliations:** Chicago


ARTICLE VI. — *Sub-involution, with Retro-flexion.* By F. B. NORCOM, M.D., Chicago.

ON 2nd January, the present year, Mrs. L——, æt. 33, a multipara, of leuco-phlegmatic temperament, came from the country and was placed under my charge, suffering from severe uterine disease of four years' standing. She had borne two children, besides having had two miscarriages, but dated her present ailment from the birth of her last child, now about four years of age. Without ever enjoying robust health, she had been always comparatively well, with normal menstruation, up to the last confinement, when, rising too early — within the first week — she began to experience pelvic pains, and leucorrhœal discharge; and, six weeks afterwards, was thrown from her carriage.

From this period she dates the aggravation of her symptoms; the pain in back, pelvis and lower abdomen, with discharge increasing, and gradually growing worse, attended by irritable bladder, more or less weight in uterine region, and depreciation of general health.

Upon visiting her, she presented a very pallid and bloodless appearance, with pinched, anxious expression; considerable emaciation; great despondency; and fear of premature death. Pulse 96, weak and irritable; tongue anæmic, and coated; bowels capricious; loss of appetite, with attendant dyspepsia (deficient gastric innervation); irregular sleep; and persistent pains in loins, pelvis, and inner thighs. The menstrual flow excessive and painful.

An examination by vagina and rectum revealed a well-marked case of retro-flexion, with moderate thickening, and acute tenderness of uterine walls, especially the posterior, which was slightly mammilated, while movement of the neck forward induced pain at the internal os. The speculum showed the canal open, and filled with tenacious mucus, neck and lips enlarged; and the sound passed in four inches without much difficulty, some degree of pain, with its convexity looking upwards, giving evidence of tenderness upon impinging upon fundus, followed by few drops of blood upon its withdrawal.

The rational and physical signs in the case rendered the diagnosis comparatively easy: sub-involution, from the early rising after delivery, and uterine atony; retro-flexion from shock, the predisposition from increased uterine weight; and concomitant sub-acute endo-metritis, with moderate degree of parenchymatous inflammation. The case was rather unpromising as to a radical cure, the more especially as the nervous system was completely shattered — a strong hysterical element predominating; the digestion much impaired; and the *morale* of the patient decidedly bad. She was very desirous of returning home, and was untiring in her persuasions to hasten the treatment.

I began by using the sponge tents, partly to secure readier access to uterine cavity, and partly for their local depletory effect; and after introducing some four or five, at intervals of few days, had well dilated the neck, and reduced the thickened condition of the walls. I now began passing, *in utero*, small suppositories of wax and cocoa butter, with morphine and iodine once a week, allowing them to remain, while I filled the *cul-de-sac* of Douglas, and enveloped the neck with iodized cotton, well rubbed in glycerine. At the same time the bowels were regulated by the "white mixture," *pro rê nata;* iron and bismuth administered; a highly nutritious diet, with wine; properly regulated exercise, on foot and in carriage; well ventilated apartments; moral suasion, etc., etc. This treatment was pursued steadily, with now and then some few alterations, and indorsed by Dr. H. W. Jones, who saw the patient several times with me, until we had reached that stage when considerable diminution of the uterine hypertrophy had taken place, and very great subsidance of pelvic and uterine tenderness. In the mean time the appetite had materially improved, the sleep undisturbed and refreshing; the anæmia much lessened, the function of hæmatosis being in better operation.

On the 15th of February the closed lever bar pessary of Hodge was introduced, placing the womb *in sitû*, with very little pain or discomfort. The cotton rubbed in glycerine wrapped around cervix and filled in behind; and in case of irritation, suppositories containing atropine and morphine, as suggested by Dr. H.W. Jones. The pessary was left in for five or six days, when she complained of so much pressure upon neck and base of bladder that I removed it; and being warmed, its anterior bar was indented and lowered in such a manner as to interfere in no wise with this important viscus. It now remained in two weeks, when, having fallen somewhat from its position, it was again removed and slightly widened, so as to further distend the vagina, and then remained to her departure from the city, March 10th. The day she left, I made a thorough examination of the parts, and found every thing in a very satisfactory condition for the time she had been under treatment; little tenderness upon palpation or movement; the thickened condition still further reduced; the sound giving no great pain; and her general health and spirits much improved.

I endeavored to impress upon her the importance of adhering to this course of treatment, and requested her to allow the pessary to remain, even should a year or more require it, until the womb had regained its normal relations, and retired properly in the pelvis.

She was informed that her period of invalidism would continue a year or so, and that she could expect no other certain or permanent relief. After her departure, I received several encouraging letters; but later, I lost all knowledge of her, and only heard within a month, that having fallen back somewhat — probably due to some neglect of the laws laid down for her guidance — she had become alarmed, and placed herself in the hands of a "magnetizer," who had removed the support, and promised to cure her by the *battery.*

